# Paraplegia in Connection with Potts' Disease

**Published:** 1911-05-06

**Authors:** Donald Armour

**Affiliations:** Surgeon to the Hospital.


### Hospital Clinics.

### PARAPLEGIA IN CONNECTION WITH POTTS' DISEASE.

By DONALD ARMOUR, F.R.C.S., Surgeon to the Hospital.

Delivered at the National Hospital for the Paralysed and Epileptic, Queen's Square, W.C.

I propose now to show you some skiagrams taken from cases which we have had in the Hospital, to show you, in the gross, the various conditions which may be seen in this way and which are responsible for the paraplegia. Here is a case in which there was a double psoas abscess, and, in addition, the paraplegia consequent upon the affection of cord and nerves. There was an abscess in the lower part of the mediastinum, with practically complete destruction of one of the bodies of the vertebræ. It was a very interesting operation, because on removing the laminæ one found the cord compressed on either side by a very definite tuberculous granuloma about the size of a bean, bilateral and symmetrical, coming through on either side and pressing on either side of the dura. It had the effect of an ordinary spinal tumour. On scraping away this granuloma one found a small opening leading from the posterior common ligament into the main abscess cavity, which occupied the mediastinum just in front of the bodies. On enlarging these openings I was able to clear out a large amount of broken-down bony debris, mixed with much tuberculous material. In that case the symptoms were due chiefly to the mechanical pressure caused by these two tubercular tumours rather than to actual disease of the meninges. The next is that of a very interesting case. Notice the large amount of destruction of bony tissue in front, with deformity. This patient was completely paraplegic; and I was asked to see the case with Dr. Buzzard, so as to come to a conclusion as to the exact nature of the condition, that is to say, whether it was not sarcoma of the spine. She had a large mass in the neck, of almost stony hardness, and there were enlarged glands. We finally concluded that it was tubercular. In spite of this large amount of destruction of bone, in spite of her advanced neurological symptoms, she recovered completely under treatment by recumbency and extension. The next picture shows the case a year later, when the condition was clearing up.

### Some Striking Conditions.

In the early part of the lecture I referred to the onset of paraplegia in relation to angular deformity and bony destruction; and I said that frequently—at this Hospital at all events—the onset of paraplegia bore no relation to the angular deformity. Here is a case in which deformity is well marked and the cord is *in situ*, a large abscess cavity extending in front of the bodies of the vertebræ, yet the patient had no symptoms of involvement of the cord at all. The next picture shows destruction of the bodies of the vertebræ, and falling back of them, with the abscess in front of the anterior common ligament, and some angular deformity in the dorso-lumbar region. Yet this patient had no cord symptoms at all. In the next picture the lesion is a little lower down; there is greater angular curvature, there is more destruction of bone, an abscess cavity in front, with bony sequestra lying in the large abscess cavity. It is an extreme degree of angular deformity; destruction and collapse of the bodies of vertebræ in front, causing bending at almost a right angle. Yet there was no compression of the cord, perhaps owing to the amount of destruction. The next also shows great destruction of bone, yet there was no involvement of nerve roots. Here is a picture of a condition in the upper cervical region, in which there was a retro-pharyngeal abscess; you can see the caseous material lying on the other side of the dura, and the dura enormously thickened in the neighbourhood, in which the symptoms due to pressure were very well marked indeed. Here is another one, which speaks for itself, in which there has been enormous destruction of the bodies, with the formation of angular deformity. You can see the thick wall of the abscess cavity, the abscess having pressed not only upon the trachea and œsophagus, pushing them forward, but during life caused pressure on the right bronchus. Here again the dura is thickened; there is a caseous mass lying behind, which no doubt was in direct connection with the original abscess cavity, and probably the cord and membranes were entirely surrounded by this caseous material and communicated at the back with another abscess lying among the muscles of the back. This is a definite degree of construction by pressure, not only due to caseous material, but also due to thickening of the dura at the site of the abscess.

This next patient was paraplegic from this displacement due to destruction of bony tissue and angular curvature, giving rise to pressure on the cord itself. The spinal canal is markedly constricted at this point, causing direct pressure by bone.

Here is one to illustrate pressure caused by sequestrum on the lower end of the cord. This illustrates the second most frequent cause of paraplegia.

### Treatment.

Time compels me to be brief in speaking of treatment. Treatment may be discussed under three heads: (1) Prophylactic treatment, (2) the so-called expectant treatment, i.e., by mechanical devices; and (3) the operative. Of these, obviously the first is that which should be aimed at, that is to say, the prevention of paraplegia by prophylactic treatment. That depends on the early diagnosis of the caries of the spine. Following upon that diagnosis there should be prolonged treatment by recumbency. If we would prevent the onset of paraplegia we must not be content with merely putting on a plaster jacket or spinal brace and allow the child to run about. It is in cases in which that is done that paraplegia supervenes. Treatment should be by rest in bed, or in some portable apparatus, for a long time, followed later by various forms of extension. The treatment by mechanical devices is called for, of course, when the paraplegia has either started or is very well established. That and the operative are the only two forms of treatment which we need consider now.

The question of how long we shall carry on the mechanical treatment, and when we shall decide on operation is one of the greatest difficulty. For there are cases in which, with even an advanced degree of paraplegia and disease of wide extent, the whole disease cleared up by applying extension after prolonged rest. But there are other cases in which rest in bed has no effect, and in which the disease can only be relieved by operation. So each case must be decided in this respect upon its own merits. There are certain contra-indications to operation. For instance, you must consider the general condition of the patient, also his surroundings. One would not advocate operation until mechanical treatment had been given a fair trial. And I think it requires a large amount of experience and practical ingenuity to carry out mechanical treatment properly. As I have said, to put on a plaster jacket, or some sort of brace, is not efficacious. Personally, I think mechanical treatment should be given a thorough and extensive trial under the best possible conditions. The worst place for this is the wards of a London hospital. The course is the same as that of tuberculosis elsewhere—I need not go into them; general hygienic conditions, with good food and cod-liver oil, and, above all, fresh air. These are as necessary for cases of paraplegia following Potts' disease as for pulmonary phthisis. Perhaps the reason we see so many cases at Queen's Square, and are called upon to operate so often, is that they come when paraplegia is established, and we have not the opportunity of treating them in the way indicated. Having tried mechanical treatment and concluded that, despite this, the disease is progressing, operation should be considered. The results from operation are not as promising as they might be, owing, I have no doubt, to the wide extent of the pathological process. Not only does that affect the nerve elements, but the bones and ligaments; and in the cases of mediastinal and psoas abscesses there is a still further extension.

The first of these slides is that of a remarkable case, on which I operated at the Belgrave Hospital for Dr. Farquhar Buzzard. It was that of a little girl, three years of age, who was completely paraplegic and in whom the disease was in the cervical region. There was destruction of almost all the cervical vertebræ. A large abscess was present, on which I came by cutting through the deep fascia at the back of the neck. The operation consisted of clearing out a lot of sequestra and bone debris. I did not think the child would live, nor that it would even get off the operating-table alive. But a perfect recovery ensued. I show you a photograph taken some months after the operation. There seems to have been a fusing together or telescoping of what was left of the cervical vertebræ into a sort of indefinite mass of bone. I show you also the picture of a case of dorsal cervical caries in a boy who was under my care, and who was completely paraplegic, with a fair degree of involvement of both arms and legs. That child recovered completely after so-called expectant treatment, and left quite well.

